# Supplementary figures and images for: Artificial intelligence and omics-based autoantibody profiling in dementia
Source: Front Immunol. 2025 May 8;16:1537659. doi: 10.3389/fimmu.2025.1537659 (PMC12095159; doi:10.3389/fimmu.2025.1537659)

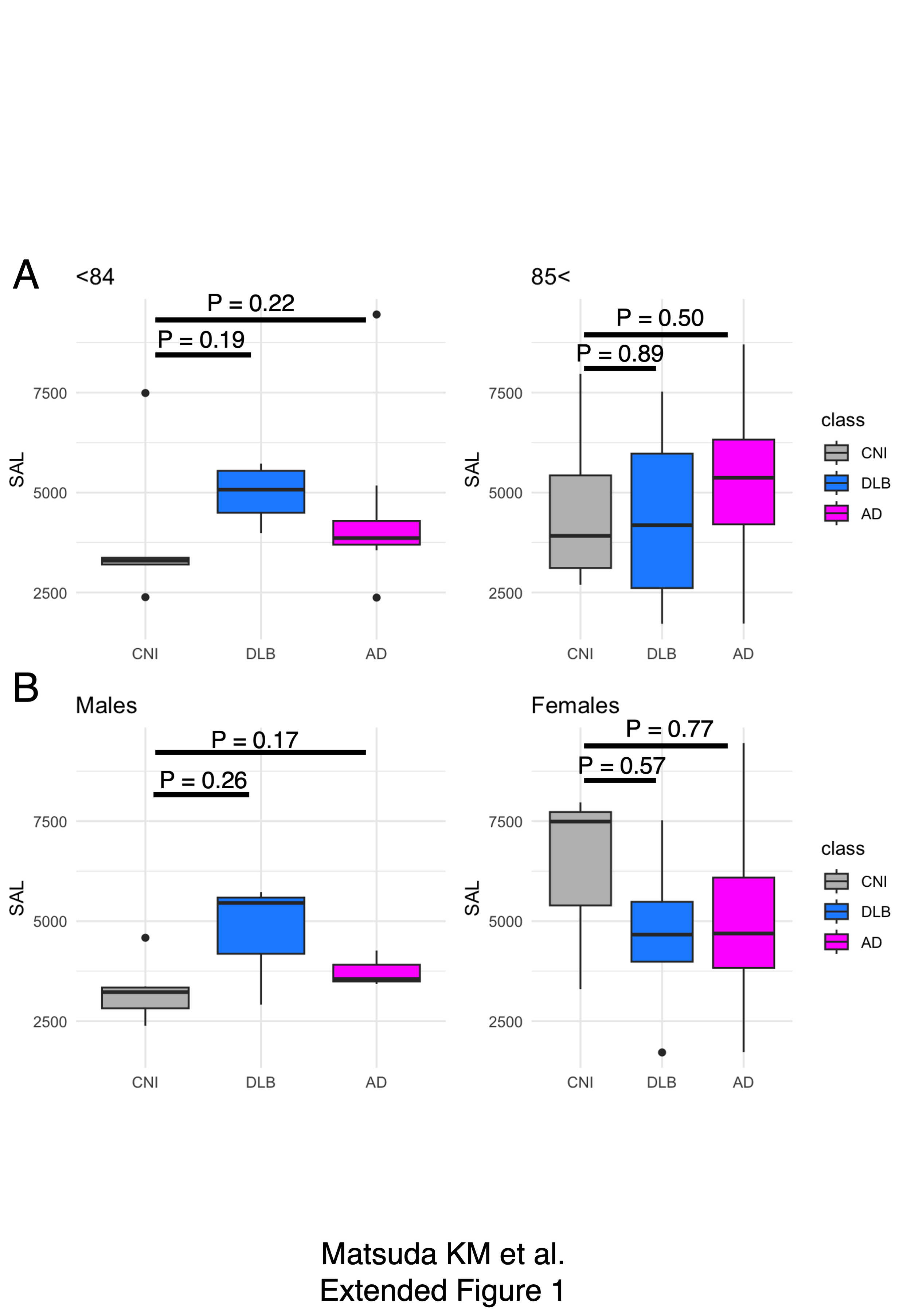

Supplement: Supplementary Figure 1 — Sum of autoantibody levels by age and sex. (A) Box plots show SAL by age groups. (B) Box plots show SAL by sex. [file Image1.tiff]

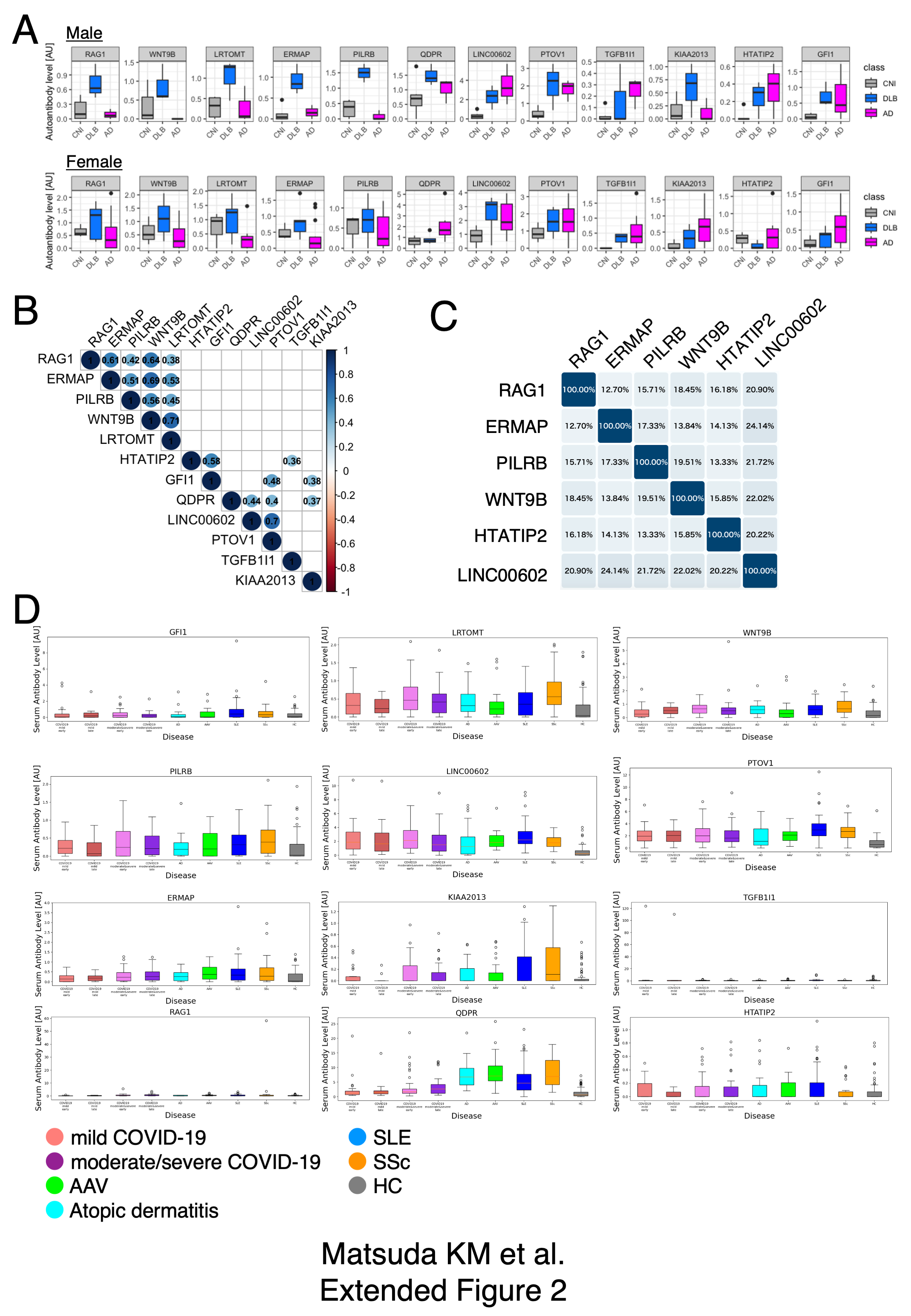

Supplement: Supplementary Figure 2 — Additional information for autoantibodies highlighted in 2-class classification tasks by AI. (A) Box plots describe the serum levels of autoantibodies highlighted in 2-class classification tasks by sex. (B) A correlation matrix of the autoantibodies highlighted in 2-class classification tasks using Spearman’s correlation. Only statistically significant pairs (P < 0.05) are shown. (C) Identity matrix, generated from aligning the corresponding protein sequences of the highly correlated autoantibodies (Spearman’s r > 0.5). (D) Box plots describe the serum levels of autoantibodies highlighted in 2-class classification tasks in COVID-19, atopic dermatitis, anti-neutrophil cytoplasmic antibody-associated vasculitis, systemic lupus erythematosus, systemic sclerosis, and healthy controls. The data derives from the UT-ABCD. [file Image2.tiff]

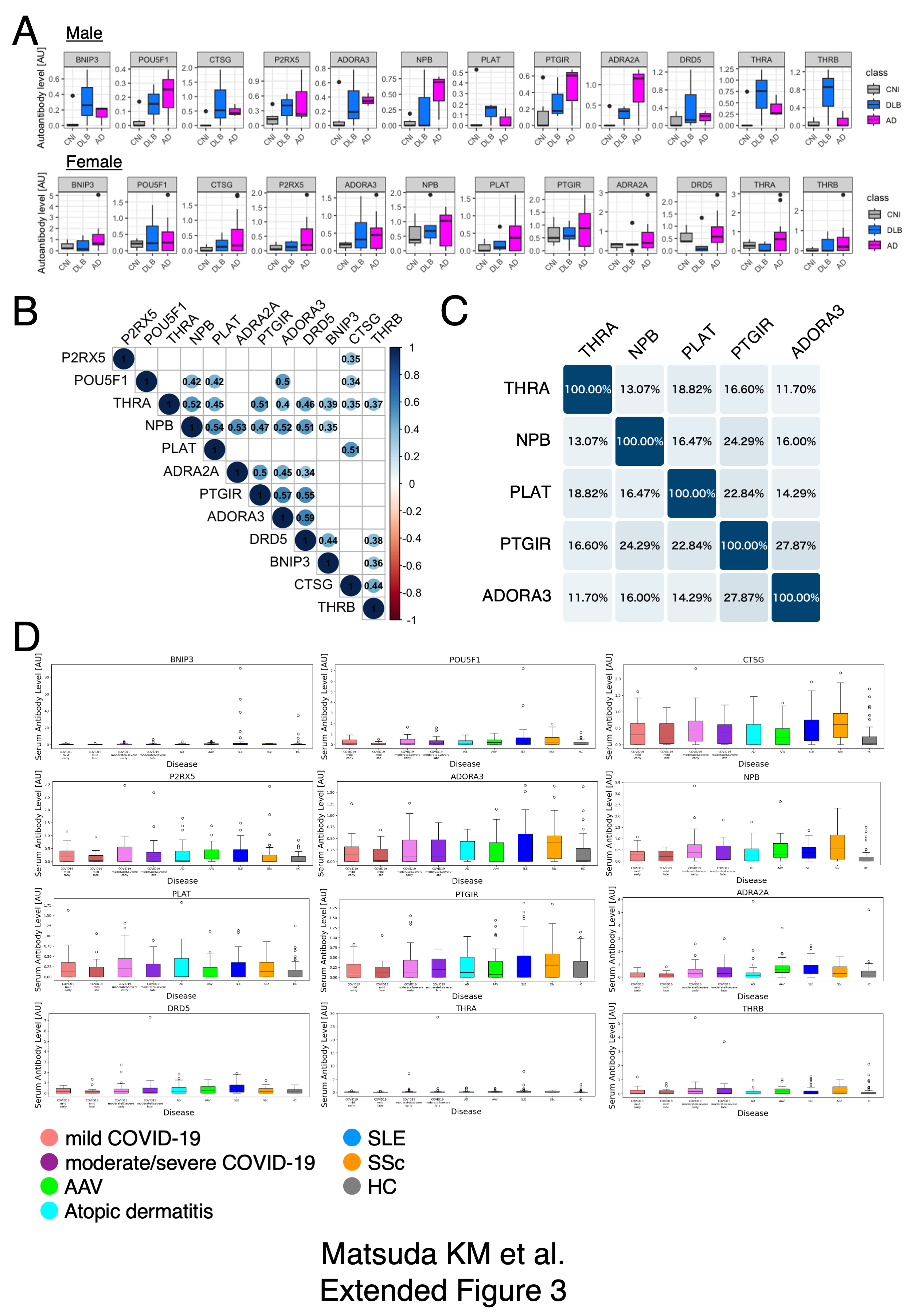

Supplement: Supplementary Figure 3 — Additional information for autoantibodies to neuroactive ligand-receptor interaction-associated proteins. (A) Box plots describe the serum levels of autoantibodies to neuroactive ligand-receptor interaction-associated proteins by sex. (B) A correlation matrix of the autoantibodies to neuroactive ligand-receptor interaction-associated proteins using Spearman’s correlation. Only statistically significant pairs (P < 0.05) are shown. (C) Identity matrix, generated from aligning the corresponding protein sequences of the highly correlated autoantibodies (Spearman’s r > 0.5). (D) Box plots describe the serum levels of autoantibodies to neuroactive ligand-receptor interaction-associated proteins in COVID-19, atopic dermatitis, anti-neutrophil cytoplasmic antibody-associated vasculitis, systemic lupus erythematosus, systemic sclerosis, and healthy controls. The data derives from the UT-ABCD. [file Image3.tiff]

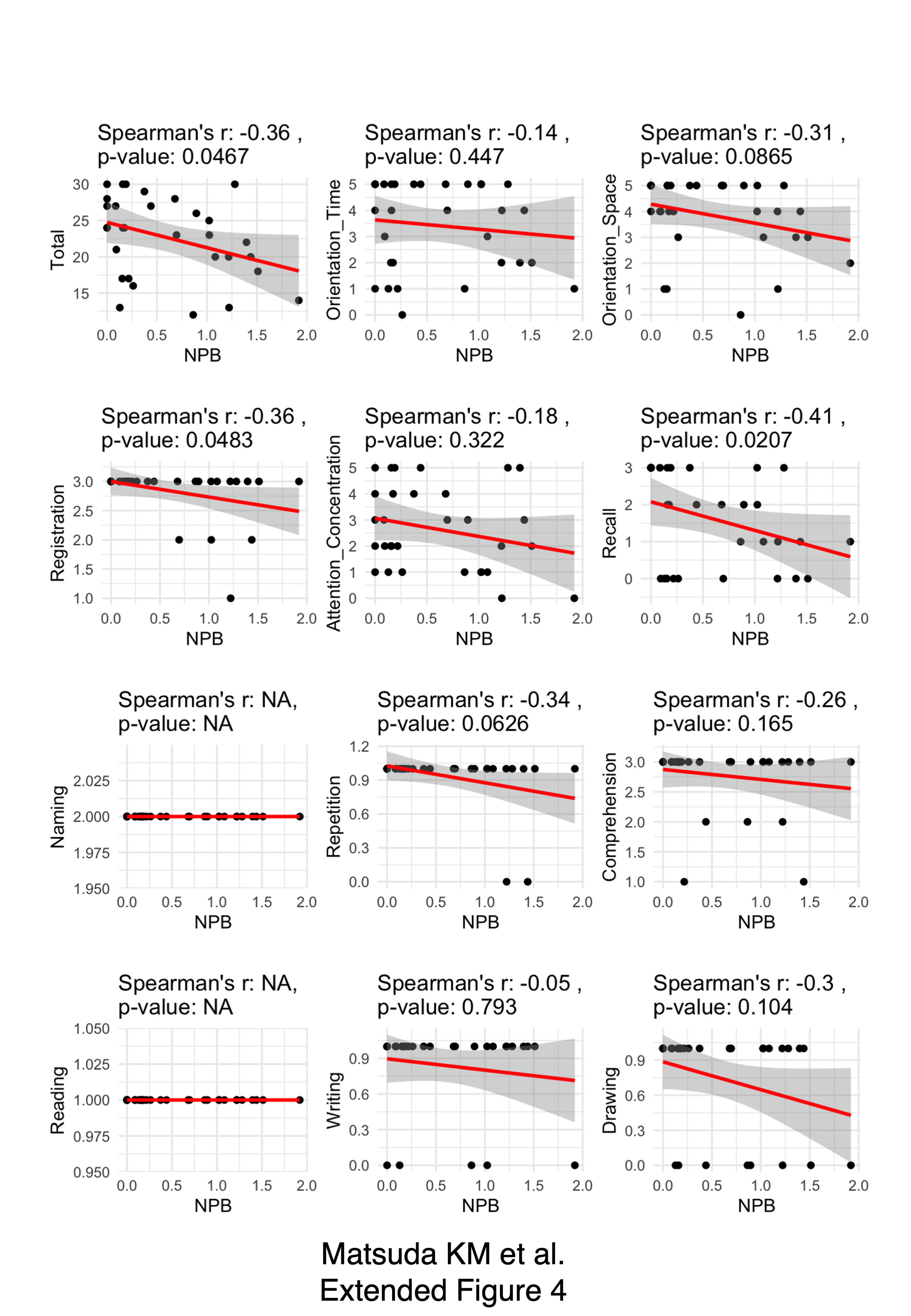

Supplement: Supplementary Figure 4 — Correlation between serum levels of anti-NPB antibodies and MMSE subscales. [file Image4.tiff]

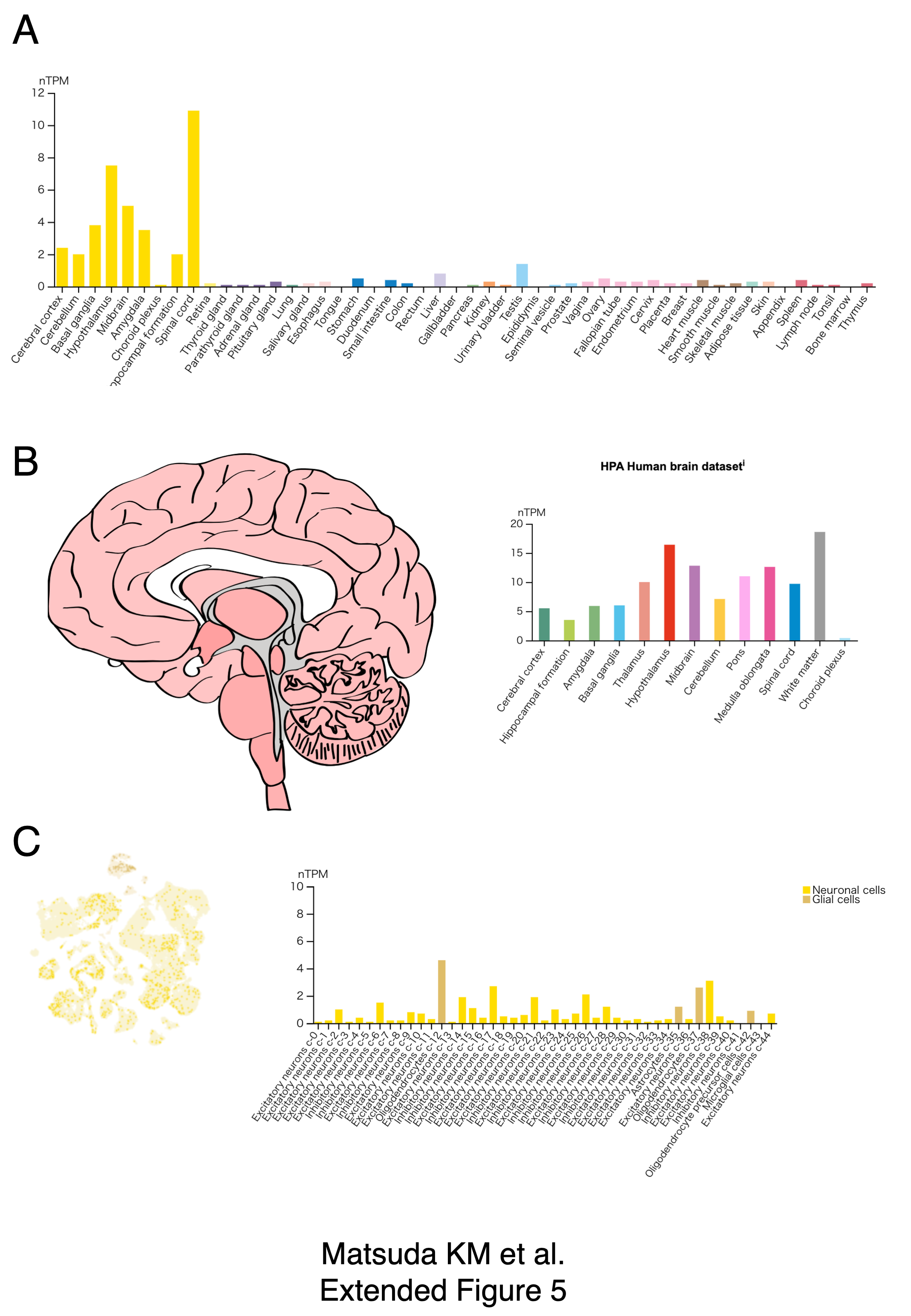

Supplement: Supplementary Figure 5 — Expression of the NPB gene in human tissues and single cells. (A) Expression of NPB in multiple human tissues measured by bulk RNA-sequencing from the Human Protein Atlas. (B) Expression of NPB in the CNS from the Human Protein Atlas. (C) Expression of NPB in the CNS evaluated by single-cell RNA-sequencing from the Human Protein Atlas. [file Image5.tiff]

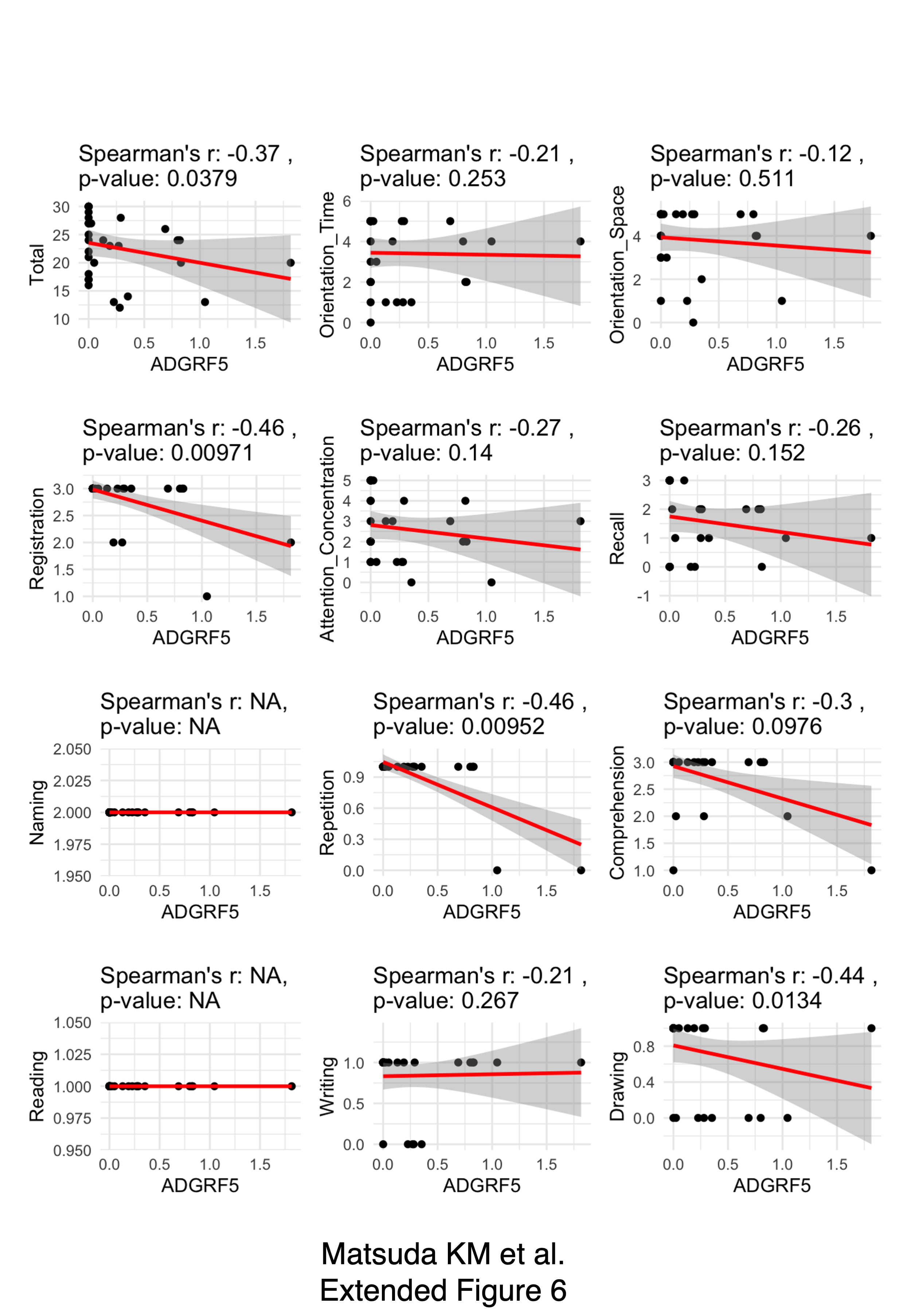

Supplement: Supplementary Figure 6 — Correlation between serum levels of anti-ADGRF5 antibodies and MMSE subscales. [file Image6.tiff]

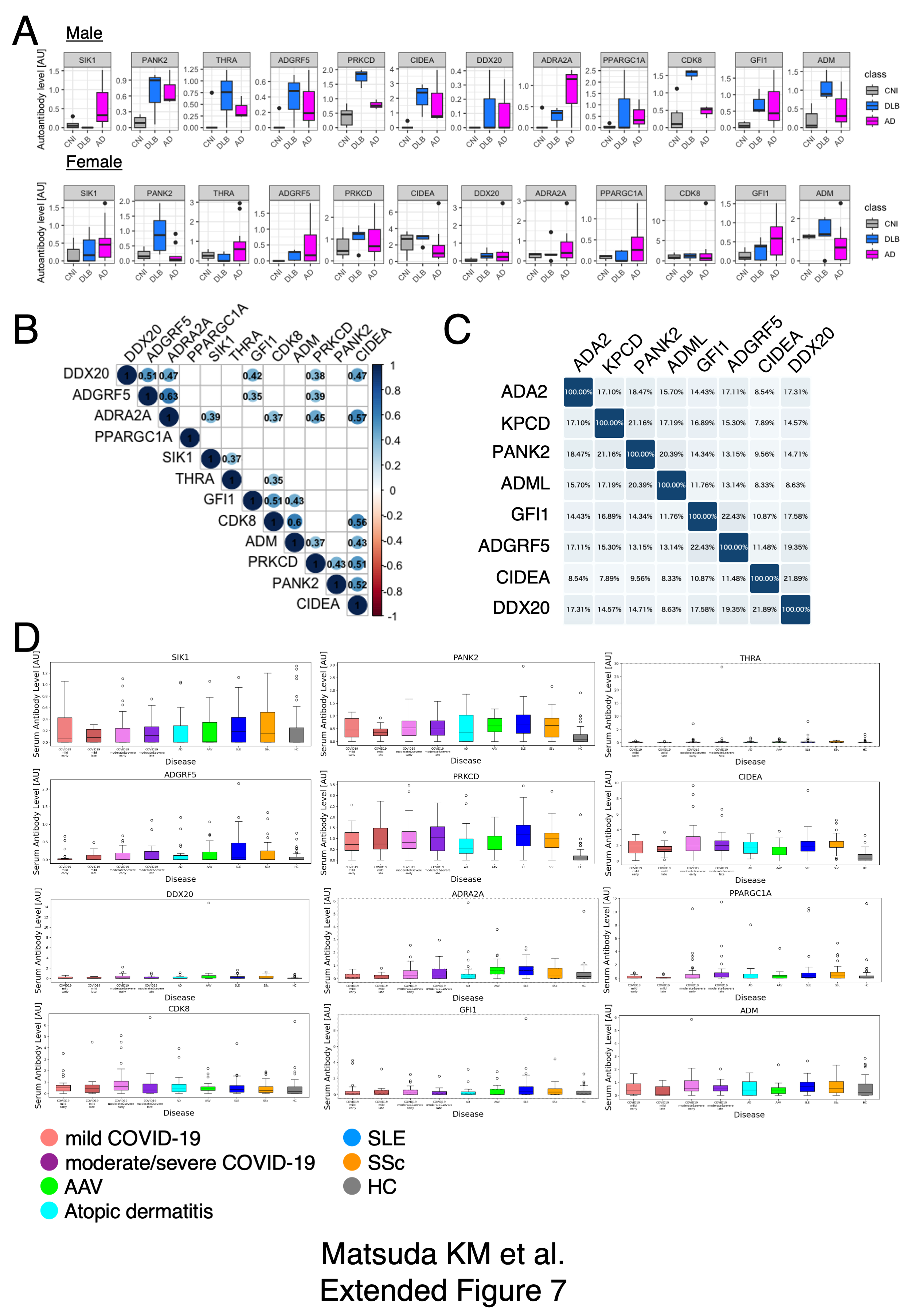

Supplement: Supplementary Figure 7 — Additional information for autoantibodies to regulation of lipid metabolic process-associated proteins. (A) Box plots describe the serum levels of autoantibodies to regulation of lipid metabolic process-associated proteins by sex. (B) A correlation matrix of the autoantibodies to regulation of lipid metabolic process-associated proteins using Spearman’s correlation. Only statistically significant pairs (P < 0.05) are shown. (C) Identity matrix, generated from aligning the corresponding protein sequences of the highly correlated autoantibodies (Spearman’s r > 0.5). (D) Box plots describe the serum levels of autoantibodies to regulation of lipid metabolic process-associated proteins in COVID-19, atopic dermatitis, anti-neutrophil cytoplasmic antibody-associated vasculitis, systemic lupus erythematosus, systemic sclerosis, and healthy controls. The data derives from the UT-ABCD. [file Image7.tiff]
